# Merkel Cell Carcinoma

**DOI:** 10.3390/biomedicines9070718

**Published:** 2021-06-23

**Authors:** Elena Dellambra, Maria Luigia Carbone, Francesca Ricci, Francesco Ricci, Francesca Romana Di Pietro, Gaia Moretta, Sofia Verkoskaia, Elisa Feudi, Cristina M. Failla, Damiano Abeni, Luca Fania

**Affiliations:** 1 Molecular and Cell Biology Laboratory, IDI-IRCCS, 00167 Rome, Italy; e.dellambra@idi.it; 2 Experimental Immunology Laboratory, IDI-IRCCS, 00167 Rome, Italy; e.feudi@idi.it (E.F.); c.failla@idi.it (C.M.F.); 3 Histopathology Unit, IDI-IRCCS, 00167 Rome, Italy; francesca.ricci@idi.it; 4 Dermatology Department, IDI-IRCCS, 00167 Rome, Italy; f.ricci@idi.it (F.R.); g.moretta@idi.it (G.M.); l.fania@idi.it (L.F.); 5 Oncology Department, IDI-IRCCS, 00167 Rome, Italy; f.dipietro@idi.it (F.R.D.P.); s.verkoskaia@idi.it (S.V.); 6 Clinical Epidemiology Unit, IDI-IRCCS, 00167 Rome, Italy; d.abeni@idi.it

**Keywords:** Merkel cell carcinoma, Merkel cell polyomavirus, immune checkpoint inhibitors

## Abstract

Merkel cell carcinoma (MCC) is a rare and extremely aggressive neuroendocrine carcinoma of the skin, with increasing incidence worldwide. This review intends to propose a comprehensive evaluation of MCC epidemiology, clinical features, pathogenetic mechanisms, diagnosis, and therapies. A section is dedicated to immunological aspects and another to the involvement of angiogenesis and angiogenic growth factors in MCC progression, proposing novel diagnostic and therapeutic approaches. Advanced MCC tumors have been treated with immune checkpoint inhibitors with effective results. Therefore, the state of art of this immunotherapy is also examined, reporting on the most recent clinical trials in the field. We conclude by underlining the achievements in the understanding of MCC pathology and indicating the present needs for effective diagnosis and therapeutic management of the disease.

## 1. Introduction

Merkel cell carcinoma (MCC) is an uncommon and extremely aggressive cutaneous neuroendocrine carcinoma [1]. MCC was first described as a “trabecular carcinoma” in 1972 by Cyril Toker [2]. The presence of neurosecretory granules was detected within the tumor cells, which were similar to those characterizing normal Merkel cells. Therefore, the tumor name was changed to MCC [3].

MCC is more common in males and elderly fair-skinned individuals subjected to chronic sun exposure. Moreover, the risk for developing MCC is increased in immunocompromised patients (e.g., chronic lymphocytic leukemia, HIV/AIDS, medical treatment for autoimmune diseases, solid organ transplantation and other types of cancers) [4,5,6].

MCC typically presents as a rapidly growing, erythematous lesion in the dermal layer of the skin [4,5,6]. The acronym AEIOU accounts for 90% of all MCC presentation: asymptomatic, expanding rapidly, immune suppression, older than 50 years, and ultraviolet (UV) radiation-exposed [7]. Besides rapid local growth, systemic tumor progression is also fast. In fact, MCC frequently metastasizes to the lymph nodes and distal organs, including liver, bone, pancreas, lung, and brain [4,5,6]. As patients are often elderly and the metastatic potential of the tumor is high, MCC prognosis is poor [4,5,6]. Thus, early diagnosis is fundamental.

There are two forms of MCC with similar presentation and prognosis but different etiology. One form is caused by integration in the patient DNA of Merkel cell polyomavirus (MCPyV) that leads to persistent expression of the viral proteins. The other form is triggered by extensive DNA mutations due to UV radiation. Both forms display immunogenic characteristics, making them interesting targets for immunotherapy [8].

Because of MCC’s aggressiveness, a multidisciplinary evaluation of patients is critical in outlining the therapeutic choice. In fact, appropriate management typically involves a combination of surgery, radiation, and chemotherapy [4,5,6]. Recently, advanced MCC has been treated with immune checkpoint inhibitors (ICI), demonstrating their remarkable efficacy [4,5,6,9,10].

In this review, we applied the same multidisciplinary approach required for effective diagnosis and therapy of tumors to provide a comprehensive evaluation of MCC’s different pathogenetic and clinical aspects. Emerging MCC diagnostic, prognostic, and therapeutic features are elaborated in depth. Finally, future perspectives for MCC investigation are proposed.

## 2. Epidemiology

The occurrence of MCC is not well-defined due to its rarity and to the scarcity of large international retrospective analyses. Generally, MCC incidence estimates are based on case studies and on single-country reviews [11]. In Europe, MCC is estimated to represent less than 1% of all cutaneous malignancies [12]. In the USA, 1500 cases of MCC are reported annually [13].

Similarly to other cancers, incidence rates of MCC in Queensland, Western Australia, and New Zealand are higher compared to other countries (age-adjusted incidence of 1.6, 0.82, and 0.88 per 100,000 per year, respectively) [14,15,16]. In Asia, incidence estimates are lower compared to other countries, and mainly based on a few published case reports [17,18].

The reported incidence of MCC is increasing over time worldwide and this could be attributed to different factors, such as improvements in cancer registration and immunohistochemical characterization and increased awareness by physicians regarding this type of cancer [19]. Specifically, in the USA, the age-adjusted incidence increased from 0.15 to 0.44 per 100,000 per year from 1986 to 2004. In addition, a Danish study reported a 5.4-fold increase from 1986 to 2003 [20,21]. Furthermore, a doubling in MCC incidence from 1998 to 2010 and from 1993 to 1997 has been reported in Germany and the Netherlands, respectively [22,23].

MCC occurrence is higher in men than in women, as reported in the USA, Germany, Australia, and New Zealand [14,22]. It is more prevalent in the white versus non-white population. Indeed, an eight-times higher age-adjusted incidence rate has been reported in white versus black populations [11,24,25]. Older people present a higher MCC risk while MCC is extremely rare in children [26,27,28]. In fact, in a population-based study from 1985 to 2013 conducted in Northeastern France, 74% of MCC cases occurred in people older than 70 years of age [29].

## 3. Clinical Features

**Figure 1:**
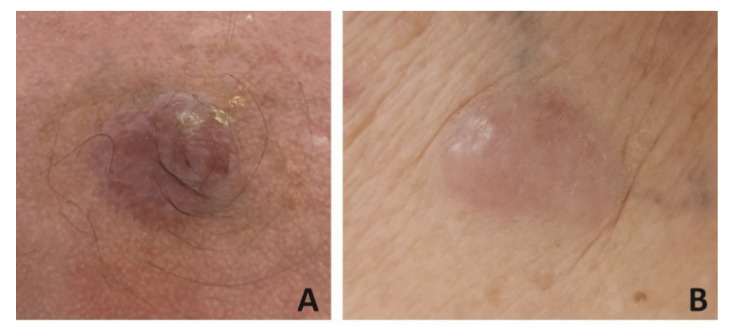
Merkel cell carcinoma (MCC) manifesting as a 3 × 3 cm firm, painless, rapidly growing, red-violet, dome-shaped, cutaneous nodule localized on the scalp in a 75-year-old organ transplant patient (**A**). MCC presenting as a 4 × 2 cm firm, painless, erythematous nodule lesion on the leg in an 82-year-old immunocompetent woman (**B**).

MCC manifests as a firm, painless, rapidly growing, red-violet, dome-shaped, cutaneous nodule. It is generally located on sun-exposed areas such as the head and neck or, less commonly, the extremities and buttocks [11,30,31] (Figure 1).

Ulceration is infrequent and MCC rarely presents with multiple lesions arising at different body sites [32]. Due to its nonspecific features, clinical diagnosis of MCC is often delayed [7]. Differential diagnosis should be posed with benign lesions (i.e., cysts, pimples, dermatofibromas, lipomas) or malignant tumors such as cutaneous squamous cell carcinoma, basal cell carcinoma, melanoma, lymphoma, malignant adnexal tumors, sebaceous carcinoma, Ewing sarcoma, and cutaneous metastasis of other tumors. The acronym AEIOU could help to guide clinicians towards the correct diagnosis, but histological examination is essential to confirm it. In the event of a solitary, firm, flesh-colored-to-red nodule with a smooth, shiny surface, generally on sun-exposed areas, clinicians should consider the possible diagnosis of MCC.

Since MCC spreads to the lymph nodes first, a sentinel lymph node biopsy (SLNB) should be performed as a staging procedure [28,33]. In fact, in certain cases, there is no evidence of the primary cutaneous tumor [19,34], and MCC is recognized only after excision of enlarged lymph nodes. In addition to regional lymph nodes, MCC metastases could be localized in the skin, distant lymph nodes, lungs, adrenal glands, pancreas, liver, brain, and bones [19].

Staging classification and prognostic stage groups for MCC are based on the American Joint Committee on Cancer (AJCC) staging system, 8th edition [35,36]. Staging classification consists of a definition of the primary tumor (T), a clinical evaluation of regional lymph nodes (clinical N), a pathological evaluation of regional lymph nodes (pathological N), and a definition of clinical and pathological distant metastasis (M). Regarding the prognostic staging, AJCC 8th edition includes six different groups (0, I, IIA, IIB, III, IV) and seven pathological stage groups (0, I, IIA, IIB, IIIA, IIIB, IV) according to the TNM classification.

Complete spontaneous MCC regressions have been rarely described. Interestingly, these cases are associated with improved prognosis [37]. In a review published in 2016, fewer than 40 cases of regression of MCC have been reported in the North American, Japanese, and European literature, even if a number of these cases have been criticized [37]. The mechanism of such a regression is unknown. However, spontaneous regressions have been reported shortly following a diagnostic biopsy or incomplete excision, suggesting that the injury can generate an inflammatory environment able to stimulate antitumor immune responses. Furthermore, MCPyV infection may promote a persistent immune-stimulatory signal in MCC cells that, in turn, became the target of the systemic host response to the virus [37]. In fact, viral positivity is associated with a better outcome in MCC [38].

## 4. Histopathological Features

Diagnosis requires microscopic evaluation as the clinical appearance is nonspecific and can mimic a variety of benign and malignant skin lesions. Distinction between primary cutaneous MCC and neuroendocrine carcinoma metastases in the skin also requires immunohistochemical and clinical pathology analysis [39].

On histological examination, MCC appears as an expansile, nodular, or diffusely infiltrative tumor within the dermis, variably in subcutis. The tumor presents a variable mixture of nodules, sheets, nests, and trabeculae of neoplastic cells. An intraepidermal component is occasionally present. MCC cells have small, round, blue tumor cells with a high nucleus–cytoplasm ratio, round/oval nuclei, finely dispersed chromatin (salt and pepper), indistinct nucleoli, and scant cytoplasm [40].

**Figure 2:**
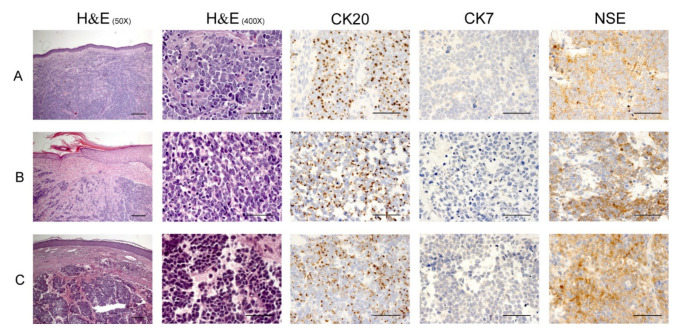
Immunohistochemistry of MCC. Panels (**A**–**C**) correspond to three representative patients. H/E staining 50× of dermal neoplastic nodules and nests. Scale bars, 200 µm. H/E 400×: same images as in 50× at higher magnification. Scale bars, 50 µm. Neoplastic cells are round, blue, with high nucleus–cytoplasm ratio, salt and pepper chromatin, and scant cytoplasm. CK20: cytoplasmatic “dot-like” CK20 positivity. CK7: MCC cells’ negativity for CK7. NSE: positivity for NSE refers to the gamma-gamma and alpha-gamma isoenzymes preferentially found in neurons and neuroendocrine cells. Scale bars, 50 µm. Gill 3 Hematoxylin (05-06015E, Bio-Optica, Milan, Italy); Eosin–Floxin (05-10020/L, Leica, Wetzlar, Germany); CK20 (clone Ks20.8, Leica, Wetzlar, Germany); CK7 (clone RN7, Leica, Wetzlar, Germany); NSE (clone 22C9, Leica, Wetzlar, Germany). Protocol FLIP BOND-III Automated IHC Stainer (Leica, Wetzlar, Germany). MCC: Merkel cell carcinoma; H/E: Hematoxylin/Eosin; CK: Cytokeratin; NSE: Neuron-specific enolase. These images are part of the diagnostic procedures in our Institute and were taken after patient written informed consent was obtained.

Mitoses and apoptotic bodies are evident, as well as nuclear molding and crush artifacts. Most cases display a pure neuroendocrine morphology, but others show divergent differentiation (e.g., squamous, sarcomatoid) [41]. MCC cells are CAM 5.2 and cytokeratin (CK) AE1/AE3 positive with a paranuclear, cytoplasmic, or mixed pattern; chromogranin, synaptophysin, CD56, neuron-specific enolase (NSE) positive, but the pathognomonic clue is CK20 positivity with a classic dot-like paranuclear pattern. MCC cells are CK7, thyroid transcription factor (TTF)-1, CDX2, S100, CD45, and vimentin negative (Figure 2). Therefore, immunohistochemistry helps in distinguishing between MCC and other skin tumors such as basal cell carcinoma (CK20^−^), lymphoma (CD45^+^), small cell melanoma (S100^+^ CK20^−^), or metastatic neuroendocrine carcinoma (CD56^+^, synaptophysin^+^ TTF-1^+^, CK20^−^) [42,43].

## 5. Dermoscopy

Dermoscopic features of MCC are not specific, but certain common characteristics can be helpful to make a differential diagnosis with other skin tumors and benign lesions. Dermoscopy of MCC reveals variable vascular patterns, such as amelanotic melanoma, predictive of malignancy. Milky red areas, associated with polymorphous, linear irregular vessels, are the most common dermoscopic features in MCC. Milky red areas are composed of areas of a slightly blurred milky red color; polymorphous vessels represent a combination of two or more different vascular patterns (dotted, glomerular, arborizing, linear) while linear irregular vessels are linear vessels with a different shape, size, and distribution [44].

The presence of these patterns and the lack of pigmentation could predict the diagnosis of MCC and prompt early management. Milky red areas are found also in benign lesions such as pyogenic granuloma, but the presence of collaret may differentiate it from MCC [45,46,47,48].

Other frequent dermoscopic characteristics exhibited in MCC are white areas, structureless areas, and architectural disorder, typical of malignant lesions. Furthermore, the absence of hyperkeratosis could separate MCC from well-differentiated squamous cell carcinoma [49].

## 6. Reflectance Confocal Microscopy (RCM)

RCM represents a useful tool for the non-invasive diagnosis of doubtful lesions [50]. RCM features for MCC have been described only in a single case report and in a recent case series [51]. RCM reveals solid aggregates of small hypo-reflective cells, resembling lymphocytes, outlined by connective fibrous tissue in the dermis. Moreover, specific polymorphic hyper-reflective cells, probably highly proliferative cells, have been observed. The epidermis appears thin and disarranged by the underlying tumors. Similar RCM features are found in amelanotic melanoma, but the presence of an evident fibrotic stroma between the aggregates of cells may help to differentiate non-melanocytic skin cancers from melanoma. In addition, in selected areas of MCC, cells are discohesive [50,51].

## 7. Pathogenesis

Two distinct etiologies are involved in the pathogenesis of MCC: i) MCPyV infection and ii) UV radiation [8,52,53]. Approximately 80% of all MCC is caused by the integration of MCPyV DNA into the host cell genome, with persistent expression of viral proteins. MCPyV-positive MCC (MCCP) tumors are characterized by a low mutational burden. Conversely, the non-viral form of MCC (MCCN) is caused by high-frequency DNA mutations following extensive UV exposition [4,8,52,53].

### 7.1. MCCP Tumors

MCPyV seroprevalence is extremely high (above 60%) in the general population and increases with age. In fact, in adults over 50 years of age, it is 80% [5,54]. MCPyV is latent in most immunocompetent subjects, but the weakening of the immune system can lead to viral reactivation [5].

MCPyV is a small, circular, double-stranded DNA virus with a genome of around 5.4 kb that consists of early and late coding regions playing a role in infectivity [55,56]. The early region encodes large and small tumor proteins (LT and ST) and a spliced form of the LT protein called 57 kT. The late gene region encodes structural capsid proteins (VP1, VP2, and VP3). In normal skin, MCPyV replicates within the nucleus using the host machinery. Viral integration is not a normal phase of its life cycle as the integrated viral DNA cannot replicate and no longer produces any viable virus [55,56].

Despite the high MCPyV seropositivity in the population, the incidence of MCC is extremely low [5,8]. Indeed, oncogenesis by MCPyV requires the rare and accidental combination of two essential events: the clonal integration of the viral DNA, including the non-coding control region, the ST and LT genes, in the cell genome, and a mutation, leading to a loss of expression of the C-terminus of the LT protein. Lack of this region inhibits viral replication. Subsequent increased synthesis of LT and ST proteins promotes cell cycle progression and survival [5]. Thus, the truncated LT may play a role in shifting the natural behavior of MCPyV from that of a replication-competent virus with virion release to an integrated pro-virus with pro-tumorigenic activity. The viral LT oncoprotein targets the tumor suppressor protein retinoblastoma (pRB), promoting cell cycle progression and contributing to uncontrolled cell proliferation [8]. ST protein binds the tumor suppressor protein phosphatase 2A and regulates the mammalian target of rapamycin pathway in mammalian cells [57]. Moreover, ST protein forms a complex with the MYC paralog MYCL (L-MYC) and its heterodimeric partner MAX [58]. In turn, ST-MYCL/MAX recruits the chromatin remodeling complex EP400 (p400) to activate downstream MYC target genes. They include factors such as MDM2, MDM4, and CK1α that assemble into a ubiquitin ligase and promote p53 degradation. Additional target genes are components of the LSD1-RCOR2-INSM1 complex, a lysine-specific demethylase, that acts as a transcriptional repressor and functions at least in part to repress ATOH1 and ncBAF complex transcriptional activity [58,59,60]. Notably, the ATOH1 gene is required for the development of Merkel cells [61,62,63]. The LSD1 complex represses ATOH1-driven expression of neural-specific genes in MCC [8,59].

The persistent expression of viral oncoproteins is necessary for the survival and progression of MCCP [64]. Mouse models indicate that ST protein is responsible for tumor development, whereas LT protein for its progression. Thus, both viral antigens are potential therapeutic targets.

### 7.2. MCCN Tumors

UV-induced tumorigenesis is characterized by a cascade of oncogenic mutations as a result of accumulating DNA damage [8]. MCCN tumors display a remarkably high tumor mutational burden, often greater than 20 somatic mutations per mega base, mainly characterized by the “UV mutational signature”, the presence of C-to-T pyrimidine dimers. Whole-exome and whole-genome sequencing reveal many somatic single-nucleotide variants, copy number alterations, and translocations [8,10,65]. In contrast, MCCP tumors exhibit near-normal diploid genomes with few somatic mutations, without a UV mutational signature, as well as few copy number amplifications, deletions, and rearrangements [8].

Loss-of-function mutations in the tumor suppressor genes *RB1* and *TP53* are predominant in MCCN tumors [8,52,65,66]. A high frequency of loss-of-function mutations in *NOTCH1,* lysine N-methyltransferase 2C (*KMT2C*), lysine N-methyltransferase 2D (*KMT2D*), and protein truncating mutations in the cadherin FAT1 gene are observed. Additional mutated or amplified genes are members of PI3K and mitogen-activated protein kinase (MAPK) pathways. For instance, activating mutations have been found in *PIK3CA* and *AKT1* whereas loss-of-function mutations in their pathway are negative regulators (*PTEN*, *TSC1*, and *TSC2*) [8,52,66]. Amplification of the *MYCL* gene is frequently observed in MCCN [8,52,66].

The genomic landscape of MCC cell lines, which are used as preclinical models in functional and pharmacological studies, has been analyzed by whole-exome sequencing. In keeping with tumor tissue data, MCCN cell lines are characterized by a high tumor mutational burden, UV-light-induced DNA damage, functionally relevant coding mutations, mainly in *RB1* and *TP53*, and large amounts of copy number variations. Conversely, MCCP cell lines display a low tumor mutational burden with few coding mutations and a lack of specific mutational signatures [65].

### 7.3. Pathways Deregulated in MCCP and MCCN

**Figure 3:**
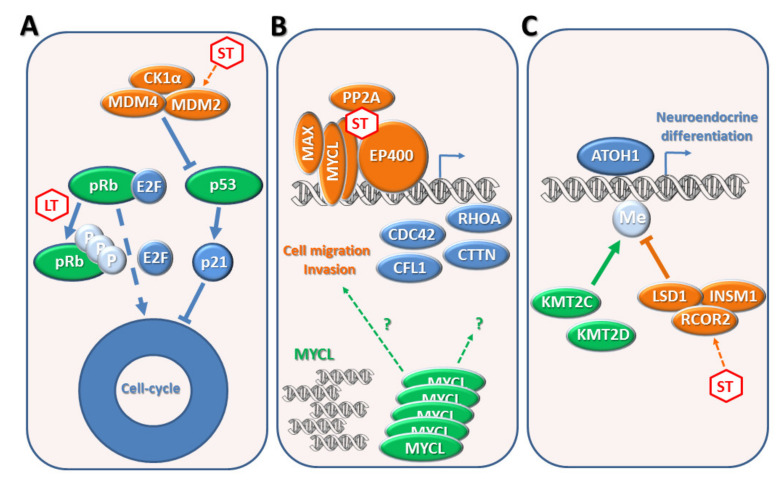
Pathways deregulated in MCCP and MCCN. (**A**) Cell cycle regulatory genes—In MCCP, MCPyV LT binds and inactivates pRB, allowing E2F-mediated transcription of S-phase cell cycle genes. The ST-MYCL-EP400 complex inactivates p53, through ubiquitin ligase (MDM2, MDM4, and CK1α) activation, determining the loss of p21 mediated cell cycle checkpoint. In MCCN, RB1 and TP53 are mutated and, in turn, the encoded proteins lose their function, promoting uncontrolled cell cycling. (**B**) MYC-dependent genes—In MCCP, the ST-MYCL-EP400 complex induces several MYC target genes, including CDC42, CFL1, CTTN, and RHOA, which are involved in cell migration and invasion. In MCCN, the MYCL gene is amplified, providing oncogenic activity, although it is not clear if MYCL acts equivalently to the ST-MYCL-EP400 complex. (**C**) The neuroendocrine differentiation program is driven by the ATOH1 transcription factor. In MCCP, the activity of the ATOH1 is partially repressed by the chromatin remodeler LSD1-RCOR2-INSM1 complex that is transactivated by the ST-MYCL-EP400 complex. LSD1 removes H3K4me2 and H3K4me1 marks, acting as a gene silencer. In MCCN, the methyltransferases KMT2D and KMT2C, which oppose LSD1 activity, are frequently mutated and therefore probably counteract ATOH1 signaling. MCCP: MCPyV-positive Merkel cell carcinoma; MCCN: non-viral form of Merkel cell carcinoma; MCPyV: Merkel cell polyomavirus; pRB: retinoblastoma protein; KMT2C: lysine N-methyltransferase 2C; KMT2D: lysine N-methyltransferase 2D; Me: methylation.

Despite the different etiologies, both forms of MCC are similar in aspect, prognosis, and response to treatment [10]. Interestingly, mutations in oncogenes and tumor suppressor genes observed in MCCN disrupt similar pathways that are targeted by the LT and ST proteins in MCCP (Figure 3) [8].

Cell cycle regulatory genes—Both forms of MCC display a high proliferative growth rate with increased levels of cell-cycle-dependent genes due to the inactivation of the tumor suppressors pRB and p53 [8,65,67] (Figure 3A). In fact, they act as cell cycle checkpoints in response to several stresses for reducing cell cycling. pRB inhibits cell cycle progression by binding to and repressing the E2F transcription factor family that activates the transcription of genes required for entry into the S phase of the cell cycle [68]. P53 controls the cell cycle mainly through direct activation of p21, which inhibits the activity of cyclin-dependent kinases CDK1 and CDK2 during the G1/S and G2/M phases [69]. MCCN tumors display a high frequency of loss-of-function mutations in the *RB1* and *TP53* genes [8]. Likewise, MCCP tumors express high levels of LT and ST proteins that inactivate pRB and p53, respectively. Although *TP53* and *RB1* are commonly wild type in MCCP, specific inactivating mutations have been reported [8,67]. In the absence of functional pRB and p53, cells can enter the S phase without any extrinsic checkpoint, inducing a deregulation of cell-cycle-dependent gene expression and high proliferative growth rate [68,69].

MYC-dependent genes—MCCP and MCCN are characterized by a strong MYC signature due to MYCL activation by the ST antigen or by gene amplification, respectively [8,58] (Figure 3B). The ST-MYCL-EP400 complex promotes the expression of several proteins of the MYC signaling pathway, including CDC42, CFL1, CTTN, and RHOA, which are involved in cell migration and invasion. It is not clear whether *MYCL* gene amplification acts equivalently to the ST-MYCL-EP400 complex activity. However, MYCL may provide a specific oncogenic activity in MCC since amplifications of MYC or MYCN genes are not commonly observed [8].

ATOH1 signaling—Both forms of MCC display an attenuated neuroendocrine differentiation program driven by the ATOH1 [8,63] (Figure 3C). Although ATOH1 is expressed in MCCP and MCCN tumors, its transcriptional activity is at least partially reduced. The ST-MYCL-EP400 complex transactivates several components of the LSD1 repressor complex. LSD1 is a chromatin remodeler that removes activating H3K4me2 and H3K4me1 marks and therefore acts as a gene silencer. ATOH1 competes with the LSD1 complex for binding to promoters of ATOH1-dependent genes in MCC cell lines. Thus, its transcriptional activity is reduced by the LSD1 complex in MCCP tumors [59].

ATOH1 levels in MCCN seem lower than those observed in MCCP. Notably, the methyltransferase activity of KMT2D and KMT2C is opposed by LSD1. Since *KMT2C* and *KMT2D* genes display inactivating mutations in MCCN tumors, their loss of function may result in reduced ATOH1 activity [8].

Other pathways—Additional signaling pathways, including PI3K Notch, Hedgehog, and bone morphogenetic protein signaling pathways, are perturbed in both MCC types [8,52,65,66]. Notably, PRC2 activity is required for the proper development of Merkel cells and may play a key role in MCC development [61,62]. PRC2 coordinated transcriptional silencing of the major histocompatibility complex class I (MHC-I) antigen processing pathway in an MCC cell line [70]. MCC tumor tissues and cell lines display low levels of MHC-I expression, which may contribute to immune evasion [71]. In a small group of MCC cases, a difference was found in the expression of the glucose transporter-1 (Glut-1), with higher expression in MCCP than in MCCN [72], indicating a possible difference in the metabolic features of the two MCC forms.

### 7.4. Cell of Origin for MCC

The cell of origin for MCC remains an open issue. Nevertheless, the cellular origin of MCC is considered a high-priority research question as it might improve preclinical studies concerning either mechanisms of tumor development or new therapeutic approaches.

Despite its name, the cells of origin of this tumor may not be Merkel cells. Although MCC and Merkel cells display similar immunophenotypes and express cytokeratin 20, synaptophysin, neural cell adhesion molecule/CD56, and numerous endocrine markers, several characteristics of MCC argue against MC as the progenitors [5,6,8,53].

A growing body of evidence indicates that MCCN tumors derive from an epidermal progenitor cell [5,6,8,53]. The high mutational burden due to UV mutations identified in MCCN is similar to the one found in keratinocyte cancers. One opposing argument against a keratinocyte origin for MCCN is the typical presentation of MCC tumors in the dermal layer. However, there are several reports of the in situ appearance of MCC associated with a cutaneous squamous cell carcinoma consistent with the possibility that MCCN can originate in the epidermal layer [8,53].

MCCP tumors seem to derive from non-epithelial cells [8,53]. Potential cells of non-epithelial origin for MCCP include B lymphocytes, due to PAX5 expression detected in certain tumors or dermal fibroblasts given how easily MCPyV replicates in this cell type [8,53]. Both cell types could also explain the typical presentation of MCC tumors in the dermal compartment. However, recent reports point to an epithelial origin also for MCCP. In fact, co-expression of viral T antigens and ATOH1 results in the development of neuroendocrine MCC-like tumors in mouse models [73,74,75,76]. Furthermore, Houben and colleagues provided clear proof that MCCP can derive from the epithelial lineage [77]. They identified the presence of an MCCP within a trichoblastoma, a benign epithelial tumor that arises from progenitor cells of the hair follicle bearing Merkel cell differentiation ability. Although MCPyV was integrated only in the MCC part of the combined tumor, six somatic mutations were shared by both tumor components. The mutational overlap between trichoblastoma and MCCP implies that MCPyV integration occurred in an epithelial tumor cell before MCC development. Therefore, hair follicles with potential for Merkel cell differentiation might represent the major cellular origin of MCCP tumors. This hypothesis is supported by rare cases of MCC found within follicular cysts and frequent connections between MCC and hair follicles [77].

## 8. Immunology

The main risk factors for the onset of MCC (i.e., age, immunosuppressive conditions, MCPyV infection, and UV exposure) are indicative of the fact that this tumor can escape an otherwise highly efficient immunosurveillance system [78]. Immune evasion relies either on changes in tumor cell populations that may acquire new characteristics or on changes in the host immune system. Both mechanisms appear relevant for MCC [79].

The high tumor mutational burden in MCCN contributes to an increased number of neoantigens that represent targets for immune T cells [8,10,80,81]. Likewise, any epitope derived from open reading frames in the viral genome may be a potential source of neoantigens in MCCP [8,10,79,80,81]. The presence of tumor-infiltrating cytotoxic CD8^+^ T lymphocytes at MCC sites indicates an active immune response to neoantigens and is associated with an improved outcome [82,83,84]. This concept has been further underlined in a recent study, where T cell receptor (TCR) sequencing was performed and the association between metrics of the TCR repertoire and MCC patient survival was measured [85]. For the examined patients, an association between the abundance of T cell clones, which reflects antigen-driven T cell expansion, and improved outcome was detected. Moreover, abundance of T cell clones, together with high CD3^+^ or CD8^+^ T cell density at the periphery of the primary MCC, constitutes a strong prognostic indicator of better survival in these patients [85].

Despite the persistent expression of immunogenic proteins, MCC may evade the immune system through several mechanisms [81]. Downregulation of MHC-I molecules, which are required for antigen presentation, occurs in 74% to 84% of MCC cases [71,86]. Interestingly, inhibitory immune molecules, including programmed death-1 (PD-1) and programmed death-ligand 1 (PD-L1), which block CD8^+^ T cell-mediated tumor cell clearance, are highly expressed in MCC samples, particularly at the tumor periphery [79,80,81,87]. Similarly, CD33^+^ myeloid-derived cells are detected at the tumor border, indicating the possible occurrence of an immune-suppressing microenvironment around MCC cells [83,87].

Failure of immune response despite the presence of CD8^+^ T cells has been found in other epithelial tumors [81]. The functional status of the immune cells or microenvironment-derived suppressive mechanisms may influence tumor progression. For instance, the significantly increased incidence of MCC with age may be due to persistent local immunosuppression by chronic UV exposure, increased incidence of immunosuppressive comorbidities, or the immune senescence process [81]. Indeed, age-related immune dysfunction leads to increased non-specific inflammatory responses, decreases CD8^+^ T cells, and impairs T cell activation. Moreover, although 92% of patients with MCC are not immunosuppressed, individuals with chronic CD8^+^ T cell dysfunction display an increased rate of MCC development [7,80].

## 9. Angiogenesis

In an attempt to identify the expression profiles associated with the aggressive behavior of MCC, Fernández-Figueras et al., using tissue microarray analyses, identified specific proteins associated with prognosis [88]. Among these proteins, and despite the weak intensity of the immunohistochemical staining, expression of vascular endothelial growth factor (VEGF)-A was associated with the more aggressive behavior of the tumor. Interestingly, VEGF-A expression was correlated with that of matrix metalloproteinases (MMP)-7 and MMP-10/2, highly involved in tumor metastasis formation. This result prompted additional investigation in the field. A study on 29 MCC patients showed that 91% were positive for VEGF-A, with a low immunohistochemical cytoplasmic staining. Furthermore, 88% of the patients were also positive for VEGF receptor (VEGFR)-2, thus supporting a possible role of this VEGF-A/VEGFR-2 angiogenic pathway in MCC pathogenesis. In addition, 75% of MCC patients were positive for VEGF-C, a growth factor specifically involved in lymph angiogenesis, and for platelet-derived growth factor (PDGF)-α, also involved in angiogenesis [89]. VEGFR-2 expression was reported in a different MCC patient cohort and a correlation between VEGFR-2 expression and tumor size was found, with larger tumors having a higher expression rate (91%) [90].

These results gave an initial indication of the molecular mechanism responsible for the high vascular density observed in certain MCC cases that was associated with lower progression-free survival (PFS) [91] and overall survival (OS) [92].

Considering the frequent involvement of lymph node metastases in MCC and the previously reported expression of the lymphangiogenic factor VEGF-C in tumor cells, lymphatic microvessel formation was examined [93]. Active intratumoral and peritumoral lymph angiogenesis was observed. On the contrary, VEGF-C expression was not reported in MCC cells, but rather in tumor-infiltrating macrophages [93].

More recently, after VEGF-A expression by immunohistochemistry was observed in 95% of MCC samples of a French cohort, Kervarrec et al. analyzed the effect of VEGF-A inhibition in a MCC mouse model [94]. A significantly lower tumor growth rate was observed in mice treated with bevacizumab, the humanized anti-human VEGF-A antibody approved for anti-angiogenic therapy in different tumor types. MCC tumor cells produced and secreted VEGF-A, especially the MCCP ones, but they did not directly respond to VEGF-A. Therefore, despite the previously described expression by MCC cells of VEGFR-2 [89,90], the role of VEGF-A inhibition in tumor growth is indirect, through blocking of angiogenesis.

VEGF-A is a central downstream factor of the hypoxia-inducible factor (HIF)-1α. Toberer et al. reported HIF-1α presence in every MCC sample that they examined, at the invading edges of the tumors, and such expression could be responsible for VEGF-A induction [72]. However, differently from what has been previously reported, they did not describe either VEGF-A or VEGFR-2 expression by MCC cells. On the other hand, they found pronounced expression of VEGFR-3, the receptor of both VEGF-A and VEGF-C.

**Figure 4:**
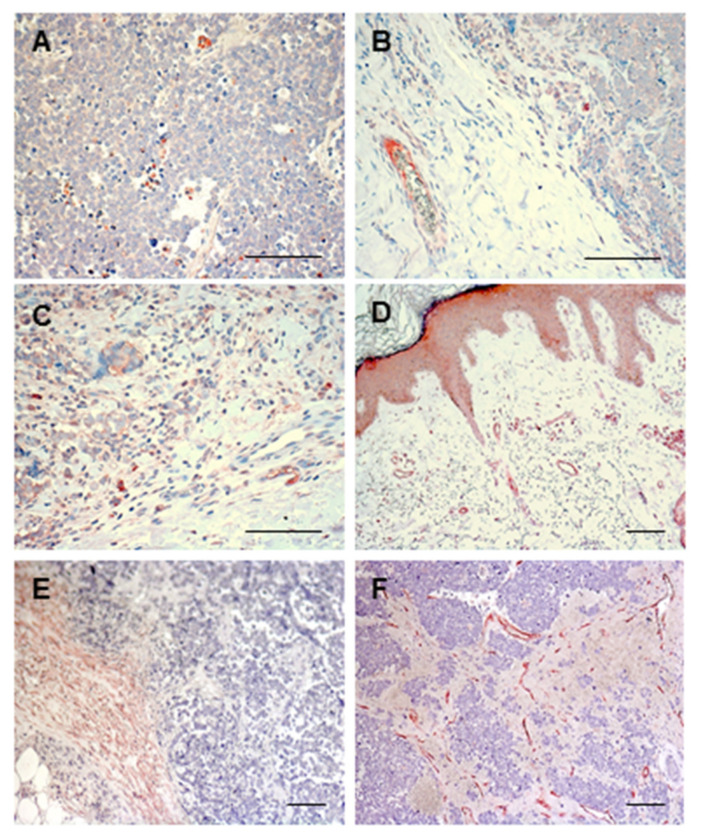
Immunohistochemistry of Merkel cell carcinoma (MCC) for angiogenic growth factor expression and vessel presence. Images from a demonstrative patient are shown. (**A**,**B**) Staining with an anti-VEGF-C antibody (clone E6, Santa Cruz Biotechnologies, Santa Crus, CA, USA), at a 1:50 dilution. Tumor and inflammatory cells are stained, as well as endothelial cells of the vessels. (**C**,**D**) Staining with an anti-VEGF-A antibody (BD Pharmingen, San Diego, CA, USA), at a 1:50 dilution. Besides tumor, inflammatory cells, and endothelial cells of the vessels, epidermal keratinocytes above the MCC lesion are also stained. (**E**) Staining with an anti-D2-40 antibody (Dako, Glostrup, Denmark), at a 1:20 dilution, to mark lymphatic vessels. (**F**) Staining with an anti-CD31/PECAM antibody (clone M20, Santa Cruz Biotechnology), at a 1:200 dilution, to show endothelial cells of both hematic and lymphatic vessels. Immunohistochemistry was performed as previously described [90], staining was done using aminoethyl carbazole (AEC, red staining), and sections were counterstained with hematoxylin. Magnification 200×, in (**A**–**C**); magnification 50×, in (**D**–**F)**; scale bars, 100 µm.

In our cases, we noticed weak cytoplasmic staining for VEGF-C in MCC cells, in a few inflammatory cells (Figure 4A), and in the endothelial cells of the vessels surrounding the tumor (Figure 4B). Specific staining for VEGF-A was also present in MCC cells and in cells of the inflammatory infiltrate (Figure 4C). As already observed by others [72], VEGF-A expression was present in the epidermis directly above the MCC lesion, and in the vessel endothelium (Figure 4D). The presence of either lymphatic or hematic vessels was detected at the tumor boundaries and inside the tumor, respectively (Figure 4E,F).

Clearly, more studies are required to explain the different data present in the literature and clearly define the role of angiogenesis, lymph angiogenesis, and of the involved growth factors in the pathogenesis of MCC.

## 10. Current Treatments

### 10.1. Surgery

Surgical excision with histologically clear margins is generally considered the treatment of choice for primary MCC without evidence of organ metastases [35,95]. Traditionally, the recommended surgical margins for primary MCC vary from 1 to 3 cm. A study on 240 patients did not demonstrate a significant difference in local recurrence or PFS between patients treated with 1, 1.1–1.9, or >2 cm margins of excision [96]. A positive surgical margin is associated with reduced OS and requires re-excision and/or adjuvant radiotherapy (RT) [97]. Given the high risk of local relapse and the importance of free excision margins, it is recommended that primary tumors should be excised with surgical margins of 1 cm (AJCC stage I) or 2 cm (AJCC stage II) [98,99,100]. Nevertheless, these studies were not randomized trials and the limited number of patients involved makes it difficult to be univocal about recommended surgical margins. In selected anatomical sites (i.e., head and neck region), smaller surgical margins may be considered to preserve anatomical function. There is no sufficient evidence to support the hypothesis that microscopically controlled surgery would yield better results for MCC. Specific retrospective studies comparing Mohs micrographic surgery (MMS) with other surgical approaches for MCC showed that MMS was associated with improved outcomes compared to standard excision [101,102]. However, other studies showed a similar rate of recurrence and OS between MMS and wide local excision with 1 to 2 cm margins. This suggests that MMS can be considered only when more exhaustive histologic margin evaluation is indicated or when tissue preservation is required [103].

### 10.2. SLNB

Given the high frequency of lymph node metastasis and the importance of lymph node staging, SLNB is highly recommended for patients without clinical and imaging evidence of metastasis. SLNB has a reported rate of positivity ranging from 30% to 38% [35,95]. However, SLNB for head and neck tumors has a higher rate of false-negative or technically unsuccessful results due to the complex lymphatic drainage. Prophylactic RT of regional nodal stations is not recommended in SLNB-negative patients, as this has not been shown to reduce the regional recurrence rate [104], but it can be indicated in patients with a negative SLNB if there is an increased recurrence risk (i.e., SLNB unsuccessful, failure to perform appropriate immunohistochemistry of SLNB, large tumor, chronic immunosuppression) [35,95].

### 10.3. Complete Lymph Node Dissection (CLND)

All patients with pathologically confirmed regional lymph node metastases should be recommended for CLND, either if it is a positive SLNB or a palpable/radiologically confirmed metastasis. However, it is currently unclear if CLND following detection of micro-metastasis in the SLNB will prolong OS [105]. In fact, recent studies report no difference in recurrence among SLNB-positive patients treated with RT or CLND [106,107]. A prospective trial examining patients treated with macroscopic (*n* = 24) and microscopic (*n* = 26) nodal disease did not show a statistically significant difference in PFS when patients were treated with definitive RT versus CLND with/without regional RT (*p* = 0.9 and *p* = 0.7, respectively) [108]. No randomized trials have compared regional nodal surgery to regional RT. Based on the above considerations, the patients with nodal involvement should be evaluated individually in multidisciplinary tumor board consultations. With respect to postoperative adjuvant RT in patients with clinically evident nodal involvement, there are data from three studies that show the benefit of additional RT of the drainage basin following CLND [109,110]. Surgical intervention is recommended for solitary organ metastases, while, in the case of multiple metastases, it is recommended to consider systemic therapies.

### 10.4. Adjuvant RT

Given the high risk of local recurrence for MCC, adjuvant RT of the tumor bed is highly recommended after surgical excision [111]. In two retrospective studies, adjuvant RT was associated with prolonged survival in patients with stage I and II disease, but not for stage III [97,112]. A retrospective analysis of 240 patients, 70% of whom received postoperative RT, reported lower local recurrence rates of 2.9%, 2.8%, and 5.2% for margins of 1 cm, 1–2 cm, and >2 cm, respectively [96]. The largest trial of 4843 MCC cases compared localized MCC (stage I and II) treated with primary surgery and adjuvant RT vs. surgery alone, showing improved OS for the first group (stage I: hazard ratio (HR) 0.71, 95% confidence interval (CI) 0.64 to 0.80, *p* < 0.001; stage II: HR 0.77, 95% CI 0.66 to 0.89, *p* < 0.001) [97]. A recent meta-analysis (29 studies, 17,179 patients) confirmed that adjuvant RT significantly improves survival (HR 0.81, *p* < 0.001) and reduces the risk of local and regional recurrence by 80% and 70%, respectively [111]. Furthermore, postoperative RT is indicated because it reduces the regional recurrence risk and improves the 3-year PFS from 48% to 76% [109]. The National Comprehensive Cancer Network (NCCN) guidelines suggest that adjuvant RT be considered in the presence of ≥1 baseline risk factor such as larger primary tumor (>2 cm diameter), chronic T-cell immunosuppression, human immunodeficiency virus infection, chronic lymphocytic leukemia, solid organ transplant, head/neck primary site, and/or lymph vascular invasion. The recommended dose is 50–60 Gy at 2 Gy/d, 5 fractions per week (F/W), with 1–2 cm margins [113]. In particular, doses of 50–56 Gy in 2 Gy fractions are recommended in cases of negative resection margins, 56–60 Gy for microscopically positive margins, and 60–66 Gy for grossly positive resection margins [114]. Adjuvant RT may be not necessary in patients with low-risk characteristics such as primary tumor < 1 cm diameter, lesion not on head/neck, negative margin status, absence of lymphatic invasion, negative SLNB, and no chronic immunosuppression [110,115]. The potential benefit of adjuvant RT should always be carefully weighed against the morbidity and frailty of the patient. Furthermore, adjuvant RT may be offered to patients who are not candidates for or refuse surgery.

### 10.5. Chemotherapy

The use of chemotherapy treatment in the adjuvant setting of MCC is controversial. There is a lack of data on survival benefits in adjuvant chemotherapy treatment in the face of significant side effects [116,117]. A retrospective study conducted on 6809 patients by the National Cancer Database showed no benefit in OS in patients with MCC, positive lymph nodes (Stage III), treated or not with adjuvant chemotherapy [97]. A prospective phase II study of 102 patients with stage I and II high-risk MCC compared adjuvant RT alone with concomitant adjuvant RT and chemotherapy with carboplatin and etoposide. Although the study was not “numerically powered” for an OS analysis, no benefit in survival was demonstrated by the addition of chemotherapy to the treatment protocol [118]. Therefore, chemotherapy is not currently considered as a therapeutic standard in the adjuvant treatment of this neoplasm and it is not routinely recommended.

### 10.6. Target Therapy

Scarce data, mainly case series, are available on the possible beneficial effects of Pazopanib and Cabozantinib, two inhibitors of multiple receptor tyrosine kinases, in the treatment of MCC. A single-arm multicenter phase II clinical trial had investigated Pazopanib in patients with metastatic MCC, and preliminary results on 16 patients showed a median PFS of 3.2 months and a median OS of 6.4 months [119]. Instead, Cabozantinib, in a prospective phase II, single-institution trial, showed low tolerability and activity in patients with platinum-failure advanced MCC [9]. Imatinib mesylate, a selective inhibitor of c-KIT, was also tested in a phase II study on 23 patients, showing a median PFS of 1 month and a median OS of 5 months [120]. Exploiting the expression of a poly ADP-ribose polymerase-1 (PARP-1) by immunohistochemistry in a fair percentage of platinum-sensitive MCC cases, the effectiveness of PARP-1 inhibitors has been taken into consideration, particularly with Olaparib, but further studies are needed to clarify the efficacy of this therapeutic approach [121].

### 10.7. Immunotherapy Clinical Trials

MCC is an immunogenic tumor, and in recent years, the introduction of a therapy with ICI has drastically improved its prognosis [122,123,124]. Currently, no indication is present for the use of ICI as adjuvant therapies, but they represent the MCC treatment of choice in the metastatic setting. Therefore, PD-1/PD-L1 inhibitors should be considered the first-line treatment of choice for patients with advanced MCC who do not have specific contraindications. The response rate to PD-1/PD-L1 inhibitors is approximately 50–70% in first line and 30% in the second or higher lines of therapy [122]. These data clearly indicate ICI’s dominance compared to chemotherapy, in which responders are fewer than 5%. Moreover, patients who initially respond to PD-1/PD-L1 inhibitors as a first- or later-line treatment have a 70–80% response durability at 2 years [122].

Pembrolizumab, an anti-PD-1 monoclonal antibody, was the first ICI to be evaluated in the treatment of MCC (Table 1). In Keynote-017, a multicenter, phase II, noncontrolled study, patients with advanced and previously untreated MCC received this agent at a dose of 2 mg/kg of body weight every 3 weeks [125]. Patients had stage IV or unresectable stage IIIB disease. In total, 26 patients were enrolled, including 17 with MCPyV-positive cancer. The objective response rate (ORR), the primary endpoint measured according to RECIST version 1.1, was 56%. Among 14 patients with an objective response, the response duration ranged from 2.2 months to at least 9.7 months, while PFS at 6 months was 67%. Tumor regression occurred also in patients with multiple organ sites and bulky disease. A rate of 15% of grade 3 and 4 adverse events (AE) has been observed [123]. Considering the encouraging results of Keynote-017, it was decided to expand the cohort to 50 patients [124]. In this study, the median duration of treatment was 6.6 months. The ORR was 56%, with 24% complete responses and 32% partial responses. An ORR of 59% was observed in virus-positive and of 53% in virus-negative tumors. Median PFS was 16.8 months, while median OS had not been reached at the time of analysis. There was no significant difference in OS or PFS between MCPyV-positive and -negative patients. PD-L1 expression, the determination of which was available in 46 patients, was not associated with response to Pembrolizumab, but a trend toward an association was observed [124].

Avelumab is a human anti-PD-L1 monoclonal antibody, evaluated in the phase II JAVELIN Merkel 200 clinical trial [79]. The 88 study participants had a diagnosis of stage IV MCC that had progressed, following ≥1 prior line of chemotherapy. Patients received Avelumab 10 mg/kg by 1 h intravenous infusion every 2 weeks. After ≥36 months of follow-up, objective responses had occurred in 33% of patients, including a complete response in 11.4%. In patients who had received 1 vs. ≥2 prior treatments, the ORRs were 40.4% and 22.2%, respectively, while, in the presence or absence of visceral metastasis, the ORRs were 34.0% and 31.7% (95% CI 18.1% to 48.1%) [126]. Based on PD-L1 expression, the ORRs were 36.8% for patients who expressed the receptor and 18.8% for the ones who did not express it. In evaluable patients with MCPyV^+^ or MCPyV^−^ tumors, the ORRs were 28.3% and 35.5%, respectively. PFS rate at 24 months was 26%, and 21% at 36 months; OS rate at 36 months was 32%, and 31% at 42 months. Median OS was higher in the PD-L1^+^ population. Treatment-related AEs of any grade occurred in 77.3% of patients. The most common AEs were fatigue, diarrhea, and nausea. Grade ≥ 3 AEs occurred in 11.4% of patients [126]. Therefore, Avelumab has been approved by both the Food and Drug Administration (FDA) and the European Medicine Agency (EMA) for the treatment of advanced MCC. The activity of the anti-cytotoxic T-lymphocyte antigen 4 (CTLA-4) Ipilimumab has also been described in five patients with metastatic MCC [127]. This agent was administered for four cycles at the dosage of 3 mg/kg every three weeks. Estimated median PFS was 12.0 months and median OS has not been reached at the time of publication. Compared to anti-PD-1 antibodies, Ipilimumab might have the advantage of a shorter treatment period, but, currently, there is insufficient evidence of its effectiveness [127].

POD1UM-201 is a phase II study in which a new anti-PD-1, INCMGA00012, is administered in MCC patients with diagnosis of metastatic disease or advanced locoregional disease, not suitable for surgery or RT and not previously treated with systemic therapies. The primary endpoint is ORR. The closure of enrollment is expected in March 2023 and preliminary results are expected to be published in the near future [128].

In addition to monotherapy, the efficacy of ICIs in combination was also evaluated. In a small retrospective study, five MCC patients were enrolled to receive the combination of the anti-PD-1 Nivolumab plus Ipilimumab as a second-line treatment. Three patients were treated with Ipilimumab 1 mg/kg plus Nivolumab 3 mg/kg and two patients with Ipilimumab 3 mg/kg plus Nivolumab 1 mg/kg. All patients previously received first-line Avelumab. The Ipilimumab/Nivolumab combination showed therapeutic activity in anti-PD-L1-refractory MCCs, but the heterogeneity of the samples, in addition to the small number of evaluated patients, did not permit any significative conclusion to be reached. The trial results should be validated on a larger cohort [129].

In another phase II randomized trial, 16 patients received as a second line of treatment Nivolumab, 240 mg every two weeks, plus Ipilimumab, 1 mg/kg every six weeks (arm A), or the same combination plus stereotactic body radiation therapy to 24 Gy in 3 fractions between cycles 1 and 2 (arm B). Out of 16 patients, one expired prior to the first evaluation. In the first analysis, it was observed that on Arm A, the response rate was 80% in 4/5 evaluable patients, while on Arm B, the response rate was 17% among six patients. Moreover, seven patients experienced >grade 2 AEs [130].

A further association under study is represented by Avelumab plus the histone deacetylase (HDAC) inhibitor Domatinostat. In the MERKLIN2 trial, a phase II study in advanced unresectable or metastatic MCC, progressed after treatment with anti-PD-1/PD-L1, the primary endpoint is ORR and the closing of the study is estimated to be in March 2024 [131] (Table 1).

As for neoadjuvant treatment, there is currently little evidence in favor of this strategy. In a recent phase I/II study conducted on 39 patients with operable MCC, Nivolumab was administered approximately 4 weeks before surgery. This study demonstrated the achievement of a pathological complete response in almost half of the patients. Regression of greater than 30% occurred in 54.5% of patients. Relapse-free survival between patients who achieved a pathological complete response and those who did not achieve one was 100% versus 59.6% at 12 months and 88.9% versus 52.2% at 24 months, respectively (HR 0.12, 95% CI 0.01–0.93). A longer follow-up is necessary to assess whether these responses translate into increased OS and further studies with a larger number of patients are essential to confirm the benefit of the neoadjuvant strategy [132]. A phase II study of Lenvatinib plus Pembrolizumab in resectable MCC is also now ongoing; the primary endpoint is the pathological complete response. Patients with stage II, III, or IV will be included (Table 1).

In the adjuvant setting, two studies are ongoing: one phase II study in which Nivolumab monotherapy will be used versus observation, in stages I-III MCC (ADMEC-O), and one pilot study in which the combination Nivolumab and Ipilimumab will be compared to Nivolumab monotherapy and RT in resected stage III MCC (NCT03798639) [133,134]. Avelumab is under investigation in two randomized trials versus placebo. A phase II study (I-MAT) in stage I–III operated MCC and a phase III study (ADAM) in stages III MCC, with affected lymph nodes, are ongoing. Another phase III study (STAMP) is underway to evaluate the efficacy of Pembrolizumab versus placebo plus possible RT after radical surgery in stages I–III MCC [133] (Table 1).

## 11. Future Perspectives

As reported above, in recent years, great achievements have been obtained for MCC. However, in our opinion, there is still room for improvement. A deeper characterization of molecular pathogenetic mechanisms and regression events would have significant repercussions for MCC management, suggesting additional treatment approaches. Downstream effectors of the ST-MYCL-EP400 complex, such as MDM2 and LSD1, could be useful for targeted therapy in MCCP tumors [8].

A deeper study on the correlation between the signal transduction effects of specific gene mutations in MCCN and the biological functions of MCPyV T antigens in MCCP may provide additional therapeutic targets and improvements on current treatments.

An aspect that has not been attentively considered so far is the metabolic reprogramming occurring in MCC [72]. As reported above, HIF-1α is highly expressed in MCC cells, and, among its downstream targets, there are genes encoding for proteins, such as glucose transporter-1 and monocarboxylate transporters, that initiate the metabolic switching from oxidative phosphorylation to glycolysis characteristic of aggressive tumor cells [135,136]. Toberer et al. found that both glucose transporter-1 and monocarboxylate transporter-4 were expressed by MCC cells and that a positive correlation between HIF-1α and glucose transporter-1 expression was present only in MCCP, suggesting a difference in the metabolic signature for the two MCC tumor types [72]. This could also explain the divergent differentiation between primary and metastatic lesions observed in one study [137].

Regarding the innovative therapeutic approaches, high expression of the somatostatin receptor in MCC has been reported, and an initial trial on 19 patients with somatostatin analogues, already used in the treatment of other neuroendocrine tumors, has been performed, with encouraging disease control as an achievement [138]. Additional studies are necessary to better evaluate this therapeutic possibility.

Even if ICI treatment can greatly improve MCC prognosis, complete and enduring responses are not satisfactorily frequent, and a great number of patients do not respond to these treatments. Combination therapies should be further considered. In this context, it should be taken into account that VEGF-A can be specifically targeted by the monoclonal antibody Bevacizumab (Avastin) and the effectiveness of this anti-angiogenic therapy has been already demonstrated in an MCC animal model [94]. Importantly, anti-VEGF-A treatment could reduce the immunosuppressive effects of VEGF-A and be used in combination with ICI immunotherapy, as proposed for other tumor types [139]. The VEGF/VEGFR pathways as well as the PDGF/PDGFR ones are also targets of the tyrosine kinase inhibitors Sorafenib (Nexavar) and Sunitinib (Sutent), successfully tested in the treatment of other tumor types [140,141,142], that could be used together with ICI as well.

## 12. Conclusions

Despite being a rare tumor, MCC has recently attracted the interest of both dermatologists and oncologists for its peculiar, not yet completely clarified pathogenesis, and for the possibility of using ICI immunotherapeutic approaches with significant positive results. Advances in understanding MCC pathogenesis have been already accomplished, and assessment of MCC responsiveness to ICI different protocols and therapeutic combinations has even now provided new treatment strategies.

Therefore, considering the increasing MCC incidence over time worldwide, it is remarkable that a new age for MCC therapy can be foreseen in the next years, opening novel perspectives and leading to additional hopes for the successful treatment of patients.

## Figures and Tables

**Table 1:** Clinical trials with immunotherapy in advanced MCC.

Trail	Study Phase	Line of Treatment	Agent	Primary Endpoint
Advanced MCC				
Keynote-017	2	First line	Pembro	ORR: 56%
JAVELIN Merkel 200	2	≥1 prior line	Avelumab	ORR: 33%
POD1UM-201	2	First line	INCMGA00012	ORR: ongoing
NCT03071406	2	≥1 prior line	IPI + Nivo + SBRT	ORR ^a^
MERKELIN2 trial	2	After anti-PD-1/PD-L1	Domat + Avelumab	ORR: ongoing
Neoadjuvant MCC				
NCT04869137	2	Neoadjuvant	Lenvatinib + Pembro	pCR
Adjuvant MCC				
ADMEC-O	2	Adjuvant, stage I–III	Nivo	PFS at 12 months
NCT03798639	1	Adjuvant, stage III	Nivo + IPI or + RT	% of patients ^b^
I-MAT	2	Adjuvant, stage I–III	Avelumab	RFS
ADAM	3	Adjuvant, stage III	Avelumab	RFS
STAMP	3	Adjuvant, stage I–III	Pembro or RT	RFS

MCC: Merkel cell carcinoma; Pembro: Pembrolizumab; IPI: Ipilimumab; Nivo: Nivolumab; SBRT: Stereotactic body radiation therapy; Domat: Domatinostat; RT: radiotherapy; ORR: objective response rate; pCR: pathological complete response; PFS: progression-free survival; RFS: relapse-free survival. ^a^ 80% arm A (IPI+Nivo) vs. 17% arm B (IPI+Nivo+SBRT). ^b^ Percentage (%) of patients completing 12 months of treatment.
